# Thinking rhythm objects

**DOI:** 10.3389/fpsyg.2022.906479

**Published:** 2022-07-15

**Authors:** Rolf Inge Godøy

**Affiliations:** ^1^Department of Musicology, University of Oslo, Oslo, Norway; ^2^RITMO Centre for Interdisciplinary Studies in Rhythm, Time and Motion, University of Oslo, Oslo, Norway

**Keywords:** rhythm, music, objects, constraints, intermittency, coherence

## Abstract

The focus of this mini-review is on *rhythm objects,* defined as strongly coherent chunks of combined sound and body motion in music, typically in the duration range of a few seconds, as may for instance be found in a fragment of dance music, in an energetic drum fill, in a flute ornament, or in a cascade of sounds of a rapid harp glissando. Although there has been much research on rhythm in continuous musical sound and its links with behavior, including the neurocognitive aspects of periodicity, synchrony, and entrainment, there has been much less focus on the generation and perception of singular coherent rhythm objects. This mini-review aims to enhance our understanding of such rhythm objects by pointing to relevant literature on coherence-enhancing elements such as *coarticulation*, i.e., the fusion of motion events into more extended rhythm objects, and *intermittent motor control,* i.e., the discontinuous, instant-by-instant control and triggering of rhythm objects.

## Introduction

We may experience music as continuous streams of sound and motion, yet also perceive music as sequences of distinct events at different timescales, be that as singular sounds, groups of sounds, motives, phrases, or more extended sections. We seem to be capable of spontaneously *parsing* sound streams (Bregman, 1990) and transforming, or *recoding*, ephemeral sensations into more solid entities in our minds by *chunking* (Miller, 1956; Gobet et al., 2016), chunking which also applies to sensations of rhythm, ending up with what we here call *rhythm objects*. *Thinking rhythm objects* is about the transition from continuous sensations to more discontinuous mental images of music-related sound and motion, and importantly, also the other way around, about generating continuous motion and sound from more solid images in our minds.

This could be seen as an *ephemeral-to-solid* (and the other way around) transition, and is a very extensive topic with ramifications to fundamental epistemological issues that were discussed by Edmund Husserl and colleagues in some remarkable publications toward the end of the 19th and beginning of the 20th century (Husserl, 1991; Godøy, 2010). Husserl’s view of perception, partly inherited from his teacher Franz Brentano, was that we need to step out of any continuous stream of sensations in order to get an overview of what we are perceiving by making some kind of cumulative and solid image of what we have been sensing. If we are continuously immersed in streams of sensations, we will not have any temporally cumulative image, i.e., not be able to have overview images, hence unable to make sense of these streams. This has later been termed by Paul Ricoeur as a need for *interrupting* continuous sensory streams in order to get an overview of what is going on (Ricoeur, 1981). Husserl came to suggest that perception proceeds by a series of such moments of cumulative overview, of what he called *now-points*, and that these now-points are composite, with traces not only of past sensations and of ongoing sensations, but importantly, also of expectations of what is to come in the future.

Significantly so, we find similar ideas of continuity-discontinuity coexistence in current theories of human motor control such as in the theory of *intermittent control,* i.e., a theory of instant-by-instant control (Craik, 1947; Gawthrop et al., 2011; Loram et al., 2011, 2014; Karniel, 2013; Sakaguchi, 2013) and in *posture-based theory,* i.e., in control by discontinuous postures (Rosenbaum, 2009, 2010, 2017; Kirsh, 2011; Warburton et al., 2013), as well as in *coarticulation*, i.e., continuous transitions between quasi-stationary postures of the effectors (vocal apparatus, fingers, and hands), where each momentary posture is conditioned by what was just done, what is done right now, and what is going to be done next (Hardcastle and Hewlett, 1999; Grafton and Hamilton, 2007; Godøy, 2014).

The core issue here is that in order to perceive any instance of rhythm, we need to perceive, and keep in memory, an extended excerpt of sound or motion. For instance, to perceive a drum pattern, we need to hear, and have some mental image of, a minimum number of successive drum sounds, just as in order to perceive a visual pattern, we need to see a minimum number of elements to decide that we actually see a visual pattern. The point of this seemingly trivial observation is to remind us that rhythm requires extension, and furthermore, that there is a qualitative transition from individual lower-level elements (i.e., individual sounds) to fused and coherent higher-level features (i.e., some pattern) in rhythm, a transition engendering features non-existent at lower levels of organization.

Yet how does the transition from an extended substrate to a compressed image of rhythm (and the other way around) work in music? This is indeed an extensive question and involves several issues of human perception and cognition; however, the focus in this mini-review will be on the role of sound-producing body motion in this transition. The main assumption here is that sound-producing motion is present in the minds and bodies of performers, but by the so-called *motor theory of perception* (Liberman and Mattingly, 1985; Galantucci et al., 2006), and by what may be called *motor-mimetic cognition* (Godøy, 2001, 2003), also present in the minds and bodies of the perceivers. Thinking rhythm as body motion objects in this way is a departure from more abstract Western notation-based concepts by studying music-related features in relation to body motion shapes (Godøy et al., 2016, 2017). To develop this line of thought in the present mini-review, we shall first have a brief overview of some main rhythm perspectives in music, followed by a section on object focus in music cognition, and sections on motor features and what may be called *quantal elements* in music, i.e., on how crucial musical features may be conceived in a discontinuous manner, before a final discussion section with some reflections on how to develop further this object-focused view of rhythm.

## Rhythm perspectives

The word “rhythm” is used with so many different significations that it may not be possible to give it a concise definition. Typically, it is taken to signify something recurrent, regular in pace, or even fluid and elegant in body motion, yet sometimes also energetic or jerky body motion. In the opinion of Shima Arom, any temporal distribution of events could conceivably be included in “rhythm” (Arom, 1992, p. 202), and with our focus on rhythm objects, adopting a general notion of rhythm makes sense, provided that occurring events can be integrated into a coherent entity, i.e., into an ‘object’.

As for the different perspectives on what musical rhythm might be, there are some instructive overview texts available (e.g., Hasty, 1997; Sethares, 2007; London, 2012). These texts also provide material on groupings of sound into what may be related to rhythm objects in our context. For instance, manifestations of meter may be instances of rhythm objects; however, our present concept of rhythm objects is more in line with Arom’s general view, and will also include complex sound masses like those of Lutoslawski (1961) or Xenakis (1992), as well as other rhythm objects in twentieth-century Western *avant garde* music (Schäffer, 1976). In particular, there are highly salient examples of rhythm objects in the theoretical and musical works of Olivier Messiaen, going back to his early work (Messiaen, 1944), including non-European music as well as bird song. In a different vein, there have been attempts to designate characteristic rhythm patterns as “signatures” of various styles (Cope, 1991), as well as more recent work with computer-assisted surveys of rhythm patterns (Cocharro et al., 2021). Furthermore, we have seen work related to gestalt phenomena (Fraisse, 1975), such as in Tenny and Polansky (1980) where coherence is associated with principles of tone proximity, as well as in work on low-level groupings of sounds based on spectral features (Bregman, 1990).

Also, we have in recent decades seen a surge in rhythm-related research in the cognitive sciences, and it seems that the boundaries between music theory and music-related neuroscience are becoming less rigid (see, e.g., London, 2012). Of particular interest here is the focus on motor components of rhythm perception and cognition playing an active role in both the generation and the perception of rhythm, be that in more periodic kinds of rhythm (Repp, 2005; Patel and Iversen, 2014; Morillon et al., 2016; Ross et al., 2016) or in more singular event-centered kinds of rhythm (Schubotz, 2007; Zimnik and Churchland, 2021). Importantly, notation-based perspectives on rhythm will have limitations in that they do not represent directly the actual output sound, and on the other hand, sound-based approaches may have difficulties in picking out groupings of rhythmic events based on sound data alone (Sethares, 2007). What is needed then is an approach that includes both sound and sound-producing motion, as these two modalities may mutually enhance the perception of coherent rhythm objects.

## Object focus

The term “object” should here be understood as designating some coherent mental entity, and not as designating something devoid of any subjective sensations. A crucial feature of “object” here is that of being present all-at-once, or in-a-now, *cf.* the mentioned idea of Husserl that perception proceeds by a series of now-points, each point encompassing a whole chunk and its internal context of recent past, present, and near future.

The main source of the object perspective here is in the theory of sound objects developed by Pierre Schaeffer and co-workers in the early days of the *musique concrète* (Godøy, 1997, 2006, 2021a; Schaeffer, 1998, 2012, 2017; Chion, 2009). Closely related to Husserl’s theories, this is an extensive theory where the main focus is on detecting and qualifying subjectively experienced features of sound objects, based on sound fragments typically in the duration range of 0.5–5 s. Any sound object should be perceived as a coherent entity, and all sequentially occurring features within the sound object will contribute to the holistic image of the sound object in question, e.g., the attack and sustain segments of a sound both contribute to the overall sensation of the sound. This object-focus grew out of the experience of the so-called “cut bell,” i.e., the removal of the attack segment of bell sounds and the resultant significant change of the perceived sound features, as well as experiences of the so-called “closed groove,” or looped sound used in the *musique concrète*, demonstrating that salient musical features are temporally distributed throughout any sound object, kept in memory as a holistic entity (Godøy, 2021a).

Furthermore, this sound object theory worked by taking sound fragments from any instrumental, vocal, environmental, or synthesis source, and then proceeding in a top-down manner by asking seemingly naïve questions as to what we are hearing, i.e., what are the shapes of the various features we are detecting. This results in the *typology* and *morphology* of sound objects, differentiating main feature dimensions such as the overall dynamic shape, overall pitch-related and/or timbral shapes, and several sub-dimensions as well as sub-sub-dimensions, all in view of highlighting esthetically salient features as shapes. Other approaches to auditory objects (e.g., Bizley and Cohen, 2013) investigate the role of top-down perceptual schemas; however, they do not go on to systematically differentiating subjectively perceived features.

Schaeffer’s typology of sound objects is based on the energy envelopes of their generation, with the main categories *impulsive*, *sustained*, and *iterative*, and reflects similar body motion categories. Also, some of the feature categories of the morphology are linked with motion features such as *gait* and *fluctuation*; hence, it made sense to extend this sound object theory to include combined sound and motion energy shapes (Godøy, 2006).

Recognizing the motor-mimetic component of music perception means that we can also recognize motor gestalts (Klapp and Jagacinski, 2011), i.e., highly coherent body motion entities, as integral to rhythm gestalts. Furthermore, the notion of motor gestalts seems to fit well with the idea of an optimal timescale for motion control in the 0.5–5 s range (Loram et al., 2014), something that in turn also seems to fit well with assumed optimal durations of sensory input in perception in the vicinity of 3 s (Pöppel, 1997), as well as with the approximate duration range of short-term memory (Snyder, 2000). Crucially, the object focus enables detecting features not found at other timescales, by enabling cumulative and/or prospective overview images of the entire rhythm object. Interestingly, similar kinds of object focus seem to be optimal also in other domains, such as in reading, with the so-called *object superiority effect* of words being easier to perceive than letters (Starrfelt et al., 2013).

## Motor features

Adopting notions of *abstract* vs. *concrete* from Pierre Schaeffer’s music theory (Schaeffer, 2017), we can classify Western music notation as abstract in the sense of generic symbols, whereas the sound-producing body motion and output sound would be concrete in the sense of continuous spatiotemporal phenomena. And although we may distinguish between sound-producing and sound-accompanying music-related body motion (Godøy and Leman, 2010), there will usually be some kind of similar motion and energy shapes between these two categories, making the motor component crucial for both production and perception of rhythm. Motor-mimetic cognition is concrete in projecting motor schemas onto sound, with motor features and sensations contributing to shaping images of sound.

One simple yet important constraint of motion and rhythm objects (as well as of gestalt perception in general, see, e.g., Bregman, 1990) is that of *exclusive allocation*, meaning here that sound-producing events cannot belong to more than one motion scheme at a time, as is the case with to so-called bistable figures (e.g., the Neckar cube or Jastrow’s duck-rabbit figure). For instance, the classic 6/8 vs. 3/4 metrical schemes can be subject to exclusive allocation in one of two gestalt figures, either as 6/8 by having two gestures with 3 eight notes in each, or as 3/4 by having three gestures with 2 eight notes in each. Also, there may be similar gestural sound event grouping in cases of polyrhythm, with concurrent rhythm motion layers becoming a single-layer motion, e.g., a three against four polyrhythm may become a monophonic series of durations (Klapp et al., 1998), and syncopations and/or offbeat events could similarly be allocated to concrete motion shapes.

Furthermore, the phenomenon of coarticulation will cause a spillover of motion from one event to another. Coarticulation happens because effector motion takes time, so that there is a constraint-based temporal smearing from one motion event to another, hence often also from one output sound to another. Most prominent in vocal performance, coarticulation can also be observed in instrumental music, enhancing coherence in the sound-producing motion as well as in the output sound, clearly contributing to the sensation of rhythm objects. Furthermore, changes in the variables of duration, rate, amplitude, and tempo may bring about changes in the degree of coarticulation (Sosnik et al., 2004; Godøy, 2014), and furthermore, changes in the degree of coarticulation may lead to changes in the category of sound-producing motion by so-called *phase transition* (Haken et al., 1985), e.g., with increased tempo from singular to fused iterative sounds.

Based on constraints of the motor control system, in particular on the so-called *psychological refractory period* entailing limitations on the ability to initiate new motion during the course of an ongoing motion (Klapp and Jagacinski, 2011), intermittent control has been seen as a workaround in that control impulses only need to occur at discontinuous points in time, with intermittent motor control variously referred to as *serial ballistic* (van de Kamp et al., 2013), *feedforward*, or *open loop*, i.e., motor control not needing continuous feedback. Related to pre-programming and hierarchization (Grafton and Hamilton, 2007), and to so-called posture-based motion (Rosenbaum, 2010, 2017), as well as so-called “marking” in dance (Kirsh, 2011; Warburton et al., 2013), one idea here is that motion can emerge by interpolating between so-called *goal postures* (Rosenbaum, 2010). Additionally, high degrees of pre-programming could also entail a fine-tuning of effort so that the force and muscular tension-relaxation activity is optimal in view of envisaged goals for the motion, as suggested by the theory of so-called *muscle synergy* (d'Avella and Lacquaniti, 2013).

## Quantal elements

Given the listed constraints of sound-producing motion in combination with constraints of perception and cognition (Godøy, 2021b), we arrive at rhythm objects as multimodal sound-motion chunks having what we could call *quantal elements* (Godøy, 2013). The term “quantal” here designates the coherent nature of a rhythm object and its associated features, containing anything between a single sound and a complex group of sounds within the confines of the rhythm object. Also, “quantal” signifies that the rhythm object is triggered by an intermittent sound-producing impulse. The point of quantal here is to recognize discontinuity as optimal for both the sound-producing motion and the coherence of the output sound object (see Godøy, 2018 for some examples), *cf.* the mentioned discontinuous now-points of Husserl.

This quantal element can make rhythm object production *divisive* (de Leeuw, 2005) in the sense that the object content stems from the spreading out of impulse energy into detailed motion events, e.g., that a single impulse may trigger an elaborate and rapid ornament, a drum fill, or any other rapid textural figure. In this way, quantal may apply also to quite complex musical material, yet at the same time optimize effort by high degrees of pre-programming, as well as enabling shifts between bursts of energy and relaxation, enabling endurance in performance, and helping avoid strain injury.

In the sense of quantal as a time-delimited sound-producing effort and time-delimited sound output, we find quantal elements in the notion of *motor gestalts* (Klapp and Jagacinski, 2011), *intermittent control* (Loram et al., 2014), and *sound objects* (Schaeffer, 2017), i.e., there are intrinsic quantal features in motion, control, triggering, and sound objects, all converging in thinking rhythm objects as coherent entities. In short, quantal elements converge in highlighting optimal features of the object timescale, i.e., ~0.5–5 s duration range, *cf.* the mentioned object superiority effect.

It remains to be further explored how such a triggering of pre-programmed motion quanta works, but so-called *startle reactions* could be interesting to explore as a general trigger of pre-planned motor chunks, and not just as a reactive pattern, *cf.* suggestions of this in (Valls-Solé et al., 1999, 2008). In our current work, we are looking at how EMG recordings combined with motion capture recordings can tell us more about the temporal aspects of impulsive motion, and EMG data from other research (Aoki et al., 1989) have suggested that shifts between effort spikes and relaxation are optimal for typically ballistic motion.

## Discussion

In summary, thinking rhythm objects means focusing on the overall shapes and perceptual features of sound and motion chunks in the 0.5–5 s range (Godøy, 2006; Schaeffer, 2017). Also, thinking rhythm objects means understanding the constraint-based unequal distribution of control and effort in music performance, i.e., understanding what may be summarized in the phenomenon of intermittency. Furthermore, intermittency may be seen as optimal in performance by enabling bypassing limitations of the psychological refractory period and by minimizing physical effort through coarticulation. Concretely, thinking rhythm objects means practicing divisive rhythm generation in the sense of top-down, hierarchical, and impulse-driven triggering of event sequences, resulting in motion within the confines of coherent rhythm objects, e.g., as ornaments, fills, ostinato figures, etc., typically in the 0.5–5 s duration range. Also, thinking rhythm objects may enhance the coherence of output sound by focusing on the overall emergent shapes of sound objects (e.g., the overall dynamic, timbral, and pitch-related envelopes). Additionally, thinking rhythm objects means exploring rhythm as sound and motion objects in the more general framework of the mentioned typological and morphological sound object features, something that could open for more cross-cultural perspectives on rhythm.

Yet, thinking rhythm objects entails some substantial challenges for future research. Firstly, there is the need to make sound-producing body motion an integral component in all kinds of rhythm research, not only in research concerned with entrainment, and secondly, to make a critical assessment of inherited Western concepts that tend to infiltrate ways of thinking rhythm as something primarily concerned with notation-related elements such as durations, meter, and metrical grids. Furthermore, thinking rhythm objects will require extensive investigations of sound-motion relationships, including developing means for correlating low-level acoustic features with more high-level typological and morphological features in analysis tools, e.g., with the *MIRtoolbox* (Lartillot and Toiviainen, 2007), and using such tools to study larger collections of rhythm objects (Godøy, 2021a). Also, we need more detailed motion capture data on sound-producing body motion to tell us more about coarticulation and posture-based motor control, as well as synced electromyographic data to tell us more about the corresponding muscular activity. Last but not least, thinking rhythm objects will require exploring intermittency in musical experience, including working toward a more fine-tuned distinction between the different timescales involved in rhythm generation and perception.

## Author contributions

The author confirms being the sole contributor of this work and has approved it for publication.

## Funding

The work on this paper has been partially supported by the Research Council of Norway through its Centres of Excellence scheme, project number 262762, and by the University of Oslo.

## Conflict of interest

The author declares that the research was conducted in the absence of any commercial or financial relationships that could be construed as a potential conflict of interest.

## Publisher’s note

All claims expressed in this article are solely those of the authors and do not necessarily represent those of their affiliated organizations, or those of the publisher, the editors and the reviewers. Any product that may be evaluated in this article, or claim that may be made by its manufacturer, is not guaranteed or endorsed by the publisher.
